# Bioactive Protopanaxatriol Type Saponins Isolated from the Roots of *Panax Notoginseng* (Burk.) F. H. Chen

**DOI:** 10.3390/molecules180910352

**Published:** 2013-08-26

**Authors:** Yi Zhang, Li-Feng Han, Kaunda Joseph Sakah, Zhi-Zhen Wu, Li-Li Liu, Kojo Agyemang, Xiu-Mei Gao, Tao Wang

**Affiliations:** 1 Tianjin State Key Laboratory of Modern Chinese Medicine, 312 Anshanxi Road, Nankai District, Tianjin 300193, China; E-Mails: zhwwxzh@hotmail.com (Y.Z.); gaoxiumei1984@hotmail.com (X.-M.G.); 2 Tianjin Key Laboratory of TCM Chemistry and Analysis, Institute of Traditional Chinese Medicine, Tianjin University of Traditional Chinese Medicine, 312 Anshan Road, Nankai District, Tianjin 300193, China; E-Mails: hanlifeng_1@163.com (L.-F.H.); zhwwxzh@263.net (J.S.S.); wuzhizhen1990@163.com (Z.-Z.W.); liulili198609@163.com (L.-L.L.); kagyemang@noguchi.mimcom.org (K.A.)

**Keywords:** *Panax notoginseng*, root, protopanaxatriol type saponins, L6 cell, mitochondrial oxidative stress

## Abstract

Seven new protopanaxatriol type saponins, 20*S*-sanchirhinosides A_1_ (**1**), A_2_ (**2**), A_3_ (**3**), A_4_ (**4**), A_5_ (**5**), and A_6_ (**6**), and sanchirhinoside B (**7**) were obtained as minor constituents from the root extract of *Panax notoginseng* (Burkill, F. H. Chen), which showed protection effects against antimycin A induced mitochondrial oxidative stress. Their structures were elucidated by chemical and spectroscopic methods (IR, HRESI-TOF-MS, 1D and 2D NMR). Among them, compounds **4**, **6** and **7** showed significant protective effects against antimycin A-induced L6 cell injury.

## 1. Introdution

Reactive oxygen species (ROS) cause protein and DNA injuries and further induce pathological changes, such as heart failure [1], neuronal injury [2] and ischemia reperfusion [3]. A lot of natural products show potential ROS scavenging effects and are used as antioxidant agents.

*Panax notoginseng* (Burkill, F. H. Chen), have been cultivated in China for more than 400 years. As a traditional Chinese medicine, whose root components have several medicinal properties and are used for stenching the blood, dispersion of gore and reduction of the pain caused by blood diseases, *etc.* The main components in this plant were identified to be saponins, flavonoids, dencichine and polysaccharides [4]. During the course of our characterization studies on the bioactive constituents from the roots of *P. notoginseng*, the 70% EtOH extract showed significant protective effects against antimycin A-induced L6 cell injuries. Seven new protopanaxatriol type saponins: 20*S*-sanchirhinosides A_1_ (**1**), A_2_ (**2**), A_3_ (**3**), A_4_ (**4**), A_5_ (**5**), and A_6_ (**6**) and sanchirhinoside B (**7**) were obtained as minor constituents from it. In this paper, we report the protect effects of *P. notoginseng* 70% EtOH extract and new compounds **1**–**7** against antimycin A-induced mitochondrial oxidative stress.

## 2. Results and Discussion

**Figure 1:**
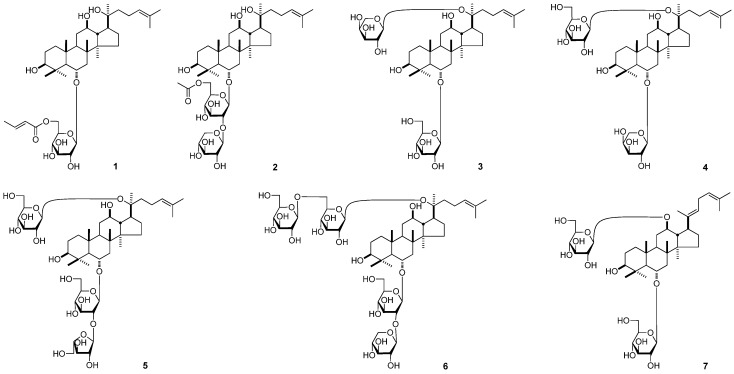
The structures of compounds **1**–**7**.

The dried roots of *P. notoginseng* were refluxed with 70% ethanol-water. Evaporation of the solvent under reduced pressure provided a 70% ethanol-water extract. The extract were subjected to column chromatography (CC) and finally HPLC to give seven new protopanaxatriol type saponins: 20*S*-sanchirhinosides A_1_–A_6_ (**1**–**6**), and sanchirhinoside B (**7**) (Figure 1).

**Figure 2:**
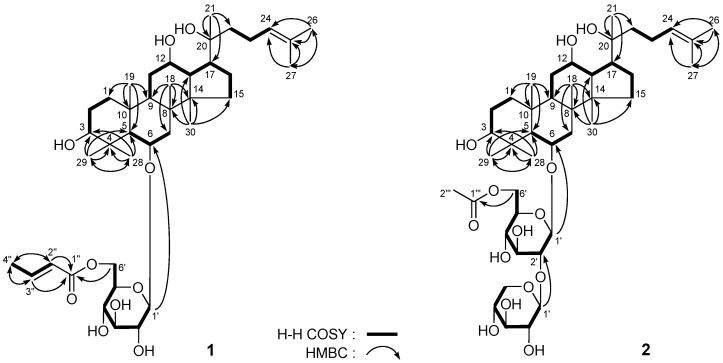
The main ^1^H ^1^H COSY and HMBC correlations of **1** and **2**.

*20(S)-Sanchirhinoside A_1_* (**1**) was isolated as a white powder, [*α*]^25^_D_ + 12.6° (MeOH). The IR spectrum showed absorption bands at 3,365, 1,717, and 1,654 cm^−^^1^ ascribable to hydroxyl, *α*,*β*-unsaturated ester, and olefin functions, respectively. The molecular formula, C_40_H_66_O_10_ of **1** was determined by positive-ion HRESI-TOF-MS (*m/z* 729.4543 [M + Na]^+^, calcd. for C_40_H_66_O_10_Na 729.4548). The ^1^H-NMR spectrum of **1** (Table 1, in C_5_D_5_N) showed signals assignable to nine methyls [*δ* 0.84, 1.07, 1.26, 1.43, 1.56, 1.64, 1.68, 2.08 (3H each, all s, H_3_-30, 19, 18, 21, 29, 27, 26, 28), 1.77 (3H, br. d, *ca*. *J* = 7 Hz, H_3_-4'')], three methines bearing oxygen functions [*δ* 3.51 (1H, dd, *J* = 5.0, 12.0 Hz, H-3), 3.93 (1H, m, H-12), 4.40 (1H, ddd, *J* = 3.5, 10.5, 10.5 Hz, H-6)], one trisubstituted olefin [*δ* 5.33 (1H, t, *J* = 7.0 Hz, H-24)], one *α*,*β*-unsaturated ester moiety [*δ* 6.06 (1H, br. d, *ca*. *J* = 16 Hz, H-2''), 7.12 (1H, dq, *J* = 7.0, 15.5 Hz, H-3'')], together with an anomeric proton signal at *δ* 5.06 (1H, d, *J* = 7.5 Hz, H-1'). The ^13^C-NMR spectrum displayed 40 carbons, including 30 carbons for the aglycon, six carbons for the sugar unit and four for a butenoyl group. Taken together the ^1^H- and ^13^C-NMR spectra suggested that **1** was a dammarane-type triterpene saponin derivative. The chemical shift of *δ*_C_ 61.5 (C-5) indicated that **1** was a protopanaxatriol type saponin [*δ*_C_~56 and ~61 (C-5) for protopanaxadiol and protopanaxatriol type saponins, respectively]. In conjunction with analysis of the HSQC spectrum, the ^1^H- and ^13^C-NMR data for **1** were assigned as shown in Table 1 (in C_5_D_5_N) and Table 2 (determined in CD_3_OD). The ^1^H ^1^H COSY experiment on **1** indicated the presence of the partial structure written in bold lines. In HMBC experiment, long-range correlations were observed between the following protons and carbons: H_3_-18 and C-7−9, 14; H_3_-19 and C-1, 5, 9, 10; H_3_-21 and C-17, 20, 22; H_3_-26 and C-24, 25, 27; H_3_-27 and C-24−26; H_3_-28 and C-3−5, 29; H_3_-29 and C-3−5, 28; H_3_-30 and C-8, 13−15; H-1' and C-6; H-6' and C-1''; H-2'', 3'' and C-1''; H_3_-4'' and C-2'', 3'' (Figure 2). The stereochemistry of C-20 in **1** was clarified by comparing the chemical shifts of 13-, 16-, 17-, and 21−24-carbons of it [*δ* 23.1 (C-23), 27.0 (C-21), 27.1 (C-16), 35.9 (C-22), 48.3 (C-13), 54.8 (C-17), 126.3 (C-24)] with those of similar 20-epimers of the dammarane type compounds, 20*R*-gensenoside Rh_1_ [*δ* 22.6 (C-21), 22.6 (C-23), 26.6 (C-16), 43.1 (C-22), 48.7 (C-13), 50.5 (C-17), 125.9 (C-24)] [5], and 20(*S*)-gensenoside Rh_1_ [*δ* 23.0 (C-23), 26.9 (C-21), 27.1 (C-16), 35.9 (C-22), 48.3 (C-13), 54.8 (C-17), 126.4 (C-24)] [6], which was measured in the same solvent (C_5_D_5_N) as **1**, the stereostructure of the 20-position in **1** was confirmed to be *S* orientation.

**Table 1:** ^1^H- and ^13^C-NMR data for compound **1** in C_5_D_5_N (500 MHz for ^1^H and 125 MHz for ^13^C).

No.	*δ*_C_	*δ*_H_ (*J* in Hz)	No.	*δ*_C_	*δ*_H_ (*J* in Hz)
1	39.5	1.05 (m), 1.74 (m)	22	35.9	1.71 (m), 2.08 (m)
2	27.9	1.85 (m), 1.90 (m)	23	23.1	2.32 (m), 2.62 (m)
3	78.7	3.51 (dd, 5.0, 12.0)	24	126.3	5.33 (t, 7.0)
4	40.3	—	25	130.8	—
5	61.5	1.43 (d, 11.5)	26	25.8	1.68 (s)
6	80.0	4.40 (ddd, 3.5, 10.5, 10.5)	27	17.7	1.64 (s)
7	45.7	1.97 (dd, 10.5, 10.5) 2.35 (m)	28	31.6	2.08 (s)
		2.52 (dd, 5.0, 10.5)	29	16.5	1.56 (s)
8	41.3	—	30	17.0	0.84 (s)
9	50.3	1.59 (m)	1'	106.2	5.06 (d, 7.5)
10	39.8	—	2'	75.4	4.06 (dd, 7.5, 9.0)
11	32.1	1.59 (m), 2.15 (m)	3'	79.2	4.22 (dd, 9.0, 9.0)
12	71.1	3.93 (m)	4'	71.6	4.00 (dd, 9.0, 9.0)
13	48.3	2.10 (dd, 10.5, 10.5)	5'	75.2	4.07 (m)
14	51.7	—	6'	65.2	4.77 (dd, 6.5, 12.0)
15	32.2	1.59 (m), 2.15 (m)			5.11 (br. d, *ca*. 12)
16	27.1	1.43 (m), 1.87 (m)	1''	166.5	—
17	54.8	2.35 (m)	2''	123.4	6.06 (br. d, *ca*. 16)
18	17.5	1.26 (s)	3''	144.8	7.12 (dq, 7.0, 15.5)
19	17.7	1.07 (s)	4''	17.9	1.77 (br. d, *ca*. 7)
20	73.0	—			
21	27.0	1.43 (s)			

**Table 2:** ^1^H- and ^13^C-NMR data for compound 1 in CD_3_OD (500 MHz for ^1^H and 125 MHz for ^13^C).

No.	*δ*_C_	*δ*_H_ (*J* in Hz)	No.	*δ*_C_^b^	*δ*_H_^b^ (*J* in Hz)
1	40.2	1.06 (m), 1.75 (m)	22	36.3	1.37 (m), 1.54 (m)
2	27.6	1.57 (m), 1.63 (m)	23	23.3	1.98 (m), 2.15 (m)
3	79.9	3.10 (dd, 5.0, 10.5)	24	126.2	5.14 (t, 7.0)
4	40.4	—	25	132.1	—
5	61.9	1.11 (d, 10.5)	26	26.0	1.68 (s)
6	80.7	4.07 (ddd, 3.0, 10.5, 10.5)	27	17.84	1.62 (s)
7	45.9	1.59 (m), 2.00 (m)	28	31.3	1.34 (s)
8	42.0	—	29	16.3	0.97 (s)
9	50.9	1.45 (m)	30	17.1	0.91 (s)
10	40.5	—	1'	105.7	4.43 (d, 7.5)
11	32.0	1.20 (m), 1.85 (m)	2'	75.5	3.21 (dd, 7.5, 9.0)
12	72.0	3.53 (m)	3'	78.7	3.35 (dd, 9.0, 9.0)
13	48.5	1.72 (dd, 11.0, 11.0)	4'	71.8	3.23 (dd, 9.0, 9.0)
14	52.5	—	5'	75.3	3.52 (m)
15	32.2	1.02 (m), 1.49 (m)	6'	65.3	4.16 (dd, 6.0, 11.5)
16	27.4	1.28 (m), 1.86 (m)			4.45 (br. d, *ca*. 12)
17	55.1	2.03 (m)	1''	168.0	—
18	17.7	1.06 (s)	2''	123.5	5.88 (dd, 2.0, 15.0)
19	17.78	0.99 (s)	3''	146.5	7.00 (dq, 7.0, 15.0)
20	74.4	—	4''	18.3	1.88 (dd, 2.0, 7.0)
21	26.5	1.15 (s) 3.63 (1H, m, overlapped)			

Acid hydrolysis yielded D-glucose, which was identified by HPLC analysis by its retention time and optical rotation using chiral detection [7,8]. On the basis of above mentioned evidence, the structure of **1** was characterized to be 20(*S*)*-*sanchirhinoside A_1_.

*20(S)-Sanchirhinoside A_2_* (**2**) was obtained as white powder with positive rotation ([*α*]_D_^25^ + 7.4°). The molecular formula, C_43_H_72_O_14_, of **2** was determined by positive-ion HRESI-TOF-MS (*m/z* 835.4832 [M + Na]^+^, calcd for C_43_H_72_O_14_Na 835.4814). Acid hydrolysis of **2** yielded D-glucose and D-xylose, which was identified by the same method as **1** [7,8]. The ^1^H and ^13^C (C_5_D_5_N, Table 3) and various 2D NMR experiments including ^1^H ^1^H COSY, HSQC, and HMBC spectra of **2** indicated the presence of a 20*S*-protopanaxatriol type aglycon [9] [*δ*_H_ 0.92, 1.00, 1.23, 1.40, 1.44, 1.64, 1.66, 2.06 (3H each, all s, H_3_-30, 19, 18, 29, 21, 27, 26, 28), 1.40 (1H, d, *J* = 11.0 Hz, H-5), 3.49 (1H, dd, *J* = 5.0, 11.5 Hz, H-3), 3.94 (1H, m, H-12), 4.34 (1H, m, H-6); *δ*_C_ 23.0 (C-23), 26.8 (C-16), 27.1 (C-21), 35.9 (C-22), 48.4 (C-13), 54.8 (C-17), 126.3 (C-24); a *β*-D-glucopyranosyl [*δ* 5.00 (1H, d, *J* = 7.5 Hz, H-1')]; a *β*-D-xylopyranosyl [*δ* 5.76 (1H, d, *J* = 7.0 Hz, H-1'')]; together with an acetyl group [*δ*_H_ 2.08 (3H, s, H_3_-2'''); *δ*_C_ 21.0 (C-2'''), 170.9 (C-1''')]. Furthermore, in the HMBC experiments, long-range correlations between the following protons and carbons were observed: H-1' and C-6; H-1'' and C-2'; H-6' and C-1'' (Figure 2). Consequently, the structure of **2** was determined and named as 20(*S*)*-*sanchirhinoside A_2_.

**Table 3:** ^1^H- and ^13^C-NMR data for compound **2** in C_5_D_5_N (500 MHz for ^1^H and 125 MHz for ^13^C).

No.	*δ*_C_	*δ*_H_ (*J* in Hz)	No.	*δ*_C_	*δ*_H_ (*J* in Hz)
1	39.5	1.00 (m), 1.70 (m)	23	23.0	2.30 (m), 2.62 (m)
2	27.8	1.85 (m)	24	126.3	5.33 (t, 7.0)
3	78.7	3.49 (dd, 5.0, 11.5)	25	130.8	—
4	40.1	—	26	25.8	1.66 (s)
5	61.2	1.40 (d, 11.0)	27	17.7	1.64 (s)
6	78.8	4.34 (m)	28	31.8	2.06 (s)
7	45.4	1.95 (dd, 10.5, 10.5) 2.35 (m)	29	17.0	1.40 (s)
		2.34 (dd, 5.0, 10.5)	30	17.0	0.92 (s)
8	41.2	—	1'	103.4	5.00 (d, 7.5)
9	50.1	1.56 (m)	2'	80.1	4.36 (dd, 7.5, 8.0)
10	39.7	—	3'	79.3	4.32 (m)
11	32.1	1.56 (m), 2.15 (m)	4'	71.3	3.99 (dd, 9.0, 9.0)
12	71.0	3.94 (m)	5'	75.0	3.94 (m)
13	48.4	2.09 (dd, 10.5, 10.5)	6'	65.0	4.61 (dd, 6.0, 11.5)
14	51.7	—			5.05 (br. d, *ca*. 12)
15	31.4	1.21 (dd, 10.0, 10.0)	1''	105.0	5.76 (d, 7.0)
		1.76 (dd, 10.0, 10.0)	2''	75.8	4.18 (m)
16	26.8	1.44 (m), 1.87 (m)	3''	78.8	4.16 (m)
17	54.8	2.34 (m)	4''	71.3	4.25 (m)
18	17.3	1.23 (s)	5''	67.3	3.66 (dd, 11.0, 11.0)
19	17.7	1.00 (s)			4.34 (m)
20	73.1	—	1'''	170.9	
21	27.1	1.44 (s) 3.63 (1H, m, overlapped)	2'''	21.0	2.08 (s)
22	35.9	1.74 (m), 2.08 (m)			

**Figure 3:**
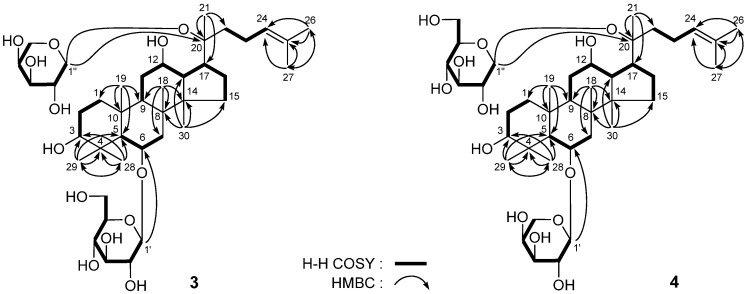
The main ^1^H ^1^H COSY and HMBC correlations of **3** and **4**.

*20(S)-Sanchirhinosides A_3_* (**3**) and *A_4_* (**4**) were both obtained as white powders with positive rotation ([*α*]_D_^25^ + 19.7° for **3**, and +23.2° for **4**, respectively, both in MeOH). The same molecular formula, C_41_H_70_O_13_, of **3** and **4** were determined by positive-ion HRESI-TOF-MS (*m/z* 793.4720 [M + Na]^+^ for **3**, 793.4715 [M + Na]^+^ for **4**, respectively, calcd for C_41_H_70_O_13_Na 793.4709). With acid hydrolysis with 1 M HCl, both of them gave D-glucose and L-arabinose [7,8]. Compared with 20*S*-gensenoside Rh_1_ [6] showed it to be similar except for the signals of an *α*-L-arabinopyranosyl moiety in the ^1^H and ^13^C (C_5_D_5_N, Table 4) data of **3** [*δ*_H_ 4.96 (1H, d, *J* = 8.0 Hz, H-1''); *δ*_C_ 66.9 (C-5''), 69.6 (C-4''), 72.6 (C-2''), 75.3 (C-3''), 98.7 (C-1'')]. On the other hand, the ^13^C-NMR chemical shift of the carbon in the 20-position was shifted from 73.0 [6] to 83.0, which indicated that C-20 was linked with a sugar. Furthermore, in the HMBC experiments, long-range correlations between H-1' and C-6, H-1'' and C-20 were observed (Figure 3). Meanwhile, the ^1^H- and ^13^C-NMR (C_5_D_5_N, Table 5) and various 2D NMR experiments including ^1^H ^1^H COSY, HSQC, and HMBC spectra of **4** showed the same fragments as **3**, including a 20*S*-protopanaxatriol type aglycon [*δ*_H_ 0.94, 1.03, 1.16, 1.48, 1.60, 1.60, 1.62, 1.98 (3H each, all s, H_3_-30, 19, 18, 29, 27, 26, 21, 28), 1.40 (1H, d, *J* = 10.5 Hz, H-5), 3.48 (1H, dd, *J* = 5.5, 10.5 Hz, H-3), 4.18 (1H, m, H-12), 4.37 (1H, m, H-6)], a *β*-D-glucopyranoside [*δ* 5.20 (1H, d, *J* = 7.5 Hz, H-1'')], and an *α*-L-arabipyranoside [*δ* 4.98 (1H, d, *J* = 8.0 Hz, H-1')]. In the HMBC experiments, long-range correlations between H-1' and C-6, H-1'' and C-20 were observed (Figure 3). On the basis of above mentioned evidence, the structures of **3** and **4** were elucidated as 20(*S*)-sanchirhinosides A_3_ and A_4_, respectively, as shown in Figure 3.

**Table 4:** ^1^H- and ^13^C-NMR data for compound **3** in C_5_D_5_N (500 MHz for ^1^H and 125 MHz for ^13^C).

No.	*δ*_C_	*δ*_H_ (*J* in Hz)	No.	*δ*_C_	*δ*_H_ (*J* in Hz)
1	39.5	1.02 (m), 1.74 (m)	23	23.2	2.22 (m), 2.50 (m)
2	28.0	1.85 (m), 1.93 (m)	24	125.9	5.28 (t, 7.0)
3	78.7	3.50 (dd, 5.0, 11.5)	25	131.1	—
4	40.4	—	26	25.8	1.62 (s)
5	61.4	1.41 (d, 10.5)	27	17.8	1.63 (s)
6	80.2	4.42 (ddd, 3.0, 10.5, 10.5)	28	31.8	2.08 (s)
7	45.2	1.94 (m), 2.50 (m)	29	16.4	1.61 (s)
8	41.1	—	30	17.2	0.81 (s)
9	50.0	1.51 (m)	1'	106.0	5.02 (d, 8.0)
10	39.7	—	2'	75.5	4.09 (dd, 8.0, 8.0)
11	31.0	1.51 (m), 2.05 (m)	3'	79.7	4.25 (m)
12	70.1	4.11 (m)	4'	71.9	4.21 (dd, 8.0, 9.0)
13	49.2	1.98 (dd, 10.5, 10.5)	5'	78.2	3.95 (m)
14	51.3	—	6'	63.1	4.37 (dd, 5.0, 12.0)
15	30.6	1.06 (m), 1.65 (m)			4.52 (dd, 1.5, 12.0)
16	26.6	1.30 (m), 1.75 (m)	1''	98.7	4.96 (d, 8.0)
17	51.5	2.48 (m)	2''	72.6	4.38 (dd, 8.0, 8.5)
18	17.60	1.17 (s)	3''	75.3	4.15 (dd, 3.0, 8.5)
19	17.55	1.03 (s)	4''	69.6	4.27 (m)
20	83.0	—	5''	66.9	3.75 (dd, 3.0, 11.0)
21	22.2	1.56 (s) 3.63 (1H, m, overlapped)			4.26 (m)
22	36.1	1.79 (m), 2.38 (m)			

**Table 5:** ^1^H- and ^13^C-NMR data for compound **4** in C_5_D_5_N (500 MHz for ^1^H and 125 MHz for ^13^C).

No.	*δ*_C_	*δ*_H_ (*J* in Hz)	No.	*δ*_C_	*δ*_H_ (*J* in Hz)
1	39.5	1.01 (m), 1.73 (m)	23	23.2	2.23 (m), 2.50 (m)
2	27.9	1.84 (m), 1.91 (m)	24	126.0	5.26 (t, 7.0)
3	78.6	3.48 (dd, 5.5, 10.5)	25	131.0	—
4	40.2	—	26	25.8	1.60 (s)
5	61.4	1.40 (d, 10.5)	27	17.8	1.60 (s)
6	79.8	4.37 (m)	28	31.7	1.98 (s)
7	45.4	1.97 (m), 2.39 (m)	29	16.6	1.48 (s)
8	41.2	—	30	17.3	0.94 (s)
9	50.0	1.55 (m)	1'	106.4	4.98 (d, 8.0)
10	39.7	—	2'	72.6	4.51 (dd, 8.0, 8.5)
11	31.0	1.55 (m), 2.09 (m)	3'	75.0	4.24 (dd, 3.0, 8.5)
12	70.2	4.18 (m)	4'	69.1	4.39 (m)
13	49.3	2.00 (dd, 10.5, 10.5)	5'	66.1	3.86 (dd, 3.0, 13.0)
14	51.4	—			4.38 (m)
15	30.8	0.99 (m), 1.61 (m)	1''	98.3	5.20 (d, 7.5)
16	26.6	1.36 (m), 1.82 (m)	2''	75.2	4.01 (dd, 7.5, 8.5)
17	51.6	2.55 (m)	3''	79.4	4.25 (dd, 8.5, 8.5)
18	17.6	1.16 (s)	4''	71.7	4.18 (dd, 8.5, 9.0)
19	17.5	1.03 (s)	5''	78.3	3.94 (m)
20	83.3	—	6''	62.9	4.34 (dd, 5.0, 11.5)
21	22.4	1.62 (s) 3.63 (1H, m, overlapped)			4.50 (dd, 1.5, 11.5)
22	36.2	1.84 (m), 2.41 (m)			

**Figure 4:**
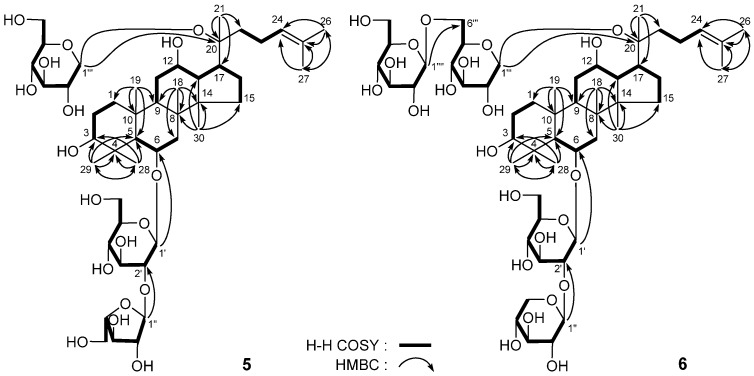
The main ^1^H ^1^H COSY and HMBC correlations of **5** and **6**.

*20(S)-Sanchirhinosides A_5_* (**5**) and *A_6_* (**6**) were both isolated as white powders with positive optical rotations ([*α*]_D_^25^ + 105.3° for **5**, and +3.1° for **6**, respectively, both in MeOH). The molecular formula, C_47_H_80_O_18_, of **5** was determined from positive-ion HRESI-TOF-MS (*m/z* 955.5248 [M + Na]^+^, calcd. for C_47_H_80_O_18_Na 955.5237). On the other hand, the molecular formula, C_53_H_90_O_23_, of **6** (*m/z* 1117.5725 [M + Na]^+^, calcd for C_53_H_90_O_23_Na 1117.5765), was determined from HRESI-TOF-MS, too. Acid hydrolysis of **5** and **6** with 1 M HCl liberated D-glucose (from **5** and **6**), D-xylose (from **6**), and L-arabinose (from **5**) [7,8]. Both the ^1^H- and ^13^C-NMR spectra of **5** and **6** (C_5_D_5_N, Table 6 for **5**, and Table 7 for **6**) indicated the presence of a 20*S*-protopanaxatriol type aglycon [9]. In conjunction with analysis of HSQC and HSQC-TOCSY spectra, the ^1^H- and ^13^C-NMR data for **5** and **6** were assigned. Meanwhile, in the HMBC experiment for compound **5**, the long-range correlations were observed between the following proton and carbon pairs: *δ*_H_ 5.10 (1H, d, *J* = 7.5 Hz, H-1') and *δ*_C_ 78.8 (C-6); *δ*_H_ 6.60 (1H, d, *J* = 2.5 Hz, H-1'') and *δ*_C_ 79.2 (C-2'); *δ*_H_ 5.16 (1H, d, *J* = 7.5 Hz, H-1''') and *δ*_C_ 83.3 (C-20) (Figure 4). On the other hand, the correlations between *δ*_H_ 4.93 (1H, d, *J* = 7.5 Hz, H-1') and *δ*_C_ 79.5 (C-6); *δ*_H_ 5.76 (1H, d, *J* = 7.0 Hz, H-1'') and *δ*_C_ 80.2 (C-2'); *δ*_H_ 5.11 (1H, d, *J* = 7.0 Hz, H-1''') and *δ*_C_ 83.5 (C-20); *δ*_H_ 5.09 (1H, d, *J* = 7.5 Hz, H-1'''') and *δ*_C_ 70.3 (C-6''') were observed in HMBC experiment on compound **6**. Consequently, compounds **5** and **6** were determined as 20(*S*)-sanchirhinosides **A_5_** and **A_6_**, respectively.

**Table 6:** ^1^H- and ^13^C-NMR data for compound **5** in C_5_D_5_N (500 MHz for ^1^H and 125 MHz for ^13^C).

No.	*δ*_C_	*δ*_H_ (*J* in Hz)	No.	*δ*_C_	*δ*_H_ (*J* in Hz)
1	39.5	0.96 (m), 1.69 (m)	26	25.8	1.61 (s)
2	27.8	1.75 (m), 1.85 (m)	27	17.8	1.61 (s)
3	78.7	3.48 (dd, 5.0, 11.5)	28	32.0	2.13 (s)
4	40.2	—	29	17.15	1.49 (s)
5	61.2	1.37 (d, 10.5)	30	17.21	0.83 (s)
6	78.8	4.41 (m)	1'	103.9	5.10 (d, 7.5)
7	45.5	1.92 (m), 2.41 (m)	2'	79.2	4.30 (dd, 7.5, 8.5)
8	41.2	—	3'	78.3	4.17 (m)
9	49.9	1.49 (m)	4'	72.0	4.17 (m)
10	39.7	—	5'	77.9	3.88 (m)
11	30.9	1.48 (m), 2.05 (m)	6'	62.8	4.32 (m)
12	70.3	4.10 (m)			4.48 (br. d, 11)
13	49.0	1.97 (dd, 10.5, 10.5)	1''	108.6	6.60 (d, 2.5)
14	51.4	—	2''	82.2	5.12 (br. s)
15	30.7	1.03 (m), 1.64 (m)	3''	77.6	4.93 (br. s)
16	26.6	1.28 (m), 1.75 (m)	4''	86.0	4.93 (br. s)
17	51.7	2.47 (m)	5''	62.4	4.18 (m)
18	17.4	1.17 (s)			4.30 (br. d, *ca*. 12)
19	17.5	0.96 (s)	1'''	98.3	5.16 (d, 7.5)
20	83.3	—	2'''	75.2	4.00 (dd, 7.5, 8.5)
21	22.4	1.60 (s) 3.63 (1H, m, overlapped)	3'''	79.2	4.24 (dd, 8.5, 8.5)
22	36.0	1.81 (m), 2.39 (m)	4'''	71.6	4.19 (dd, 8.5, 9.0)
23	23.3	2.25 (m), 2.50 (m)	5'''	78.3	3.92 (m)
24	126.0	5.27 (t, 7.0)	6'''	62.9	4.32 (m)
25	131.0	—			4.48 (br. d, *ca*. 11)

**Table 7:** ^1^H- and ^13^C-NMR data for compound 6 in C_5_D_5_N (500 MHz for ^1^H and 125 MHz for ^13^C).

No.	*δ*_C_	*δ*_H_ (*J* in Hz)	No.	*δ*_C_	*δ*_H_ (*J* in Hz)
1	39.5	0.94 (m), 1.71 (m)	1'	103.6	4.93 (d, 7.5)
2	27.8	1.81 (m)	2'	80.2	4.39 (dd, 7.5, 8.5)
3	78.9	3.48 (dd, 5.0, 11.0)	3'	79.9	4.35 (dd, 8.5, 8.5)
4	40.2	—	4'	71.8	4.18 (m)
5	61.3	1.37 (d, 10.0)	5'	78.0	3.83 (m)
6	79.5	4.32 (m)	6'	62.9	4.31 (m)
7	45.0	1.93 (m), 2.35 (m)			4.57 (br. d, *ca*. 11)
8	41.2	—	1''	104.9	5.76 (d, 7.0)
9	50.0	1.48 (dd, 11.0, 11.0)	2''	75.9	4.16 (dd, 7.0, 8.5)
10	39.7	—	3''	78.8	4.25 (m)
11	30.9	1.50 (m), 2.04 (m)	4''	71.3	4.25 (m)
12	70.2	4.16 (m)	5''	67.3	3.66 (dd, 10.5, 10.5)
13	49.2	1.98 (dd, 10.5, 10.5)			4.33 (m)
14	51.4	—	1'''	98.1	5.11 (d, 7.0)
15	30.7	1.07 (m), 1.61 (m)	2'''	74.9	3.90 (dd, 7.0, 9.0)
16	26.6	1.28 (m), 1.72 (m)	3"'	79.3	4.17 (m)
17	51.6	2.51 (m)	4'''	71.6	4.05 (m)
18	17.59	1.15 (s)	5'''	77.1	4.06 (m)
19	17.55	0.97 (s)	6'''	70.3	4.31 (m)
20	83.5	—			4.72 (br. d, *ca*. 11)
21	22.3	1.63 (s) 3.63 (1H, m, overlapped)	1''''	105.4	5.09 (d, 7.5)
22	36.2	1.80 (m), 2.40 (m)	2''''	75.3	4.04 (m)
23	23.2	2.39 (m), 2.60 (m)	3"''	78.36	4.21 (m)
24	126.0	5.32 (t, 7.0)	4''''	71.7	4.21 (m)
25	131.1	—	5''''	78.41	3.92 (m)
26	25.8	1.61 (s)	6''''	62.8	4.36 (m)
27	18.0	1.67 (s)			4.51 (br. d, *ca*. 12)
28	31.7	2.06 (s)			
29	16.7	1.46 (s)			
30	17.2	0.80 (s)			

**Figure 5:**
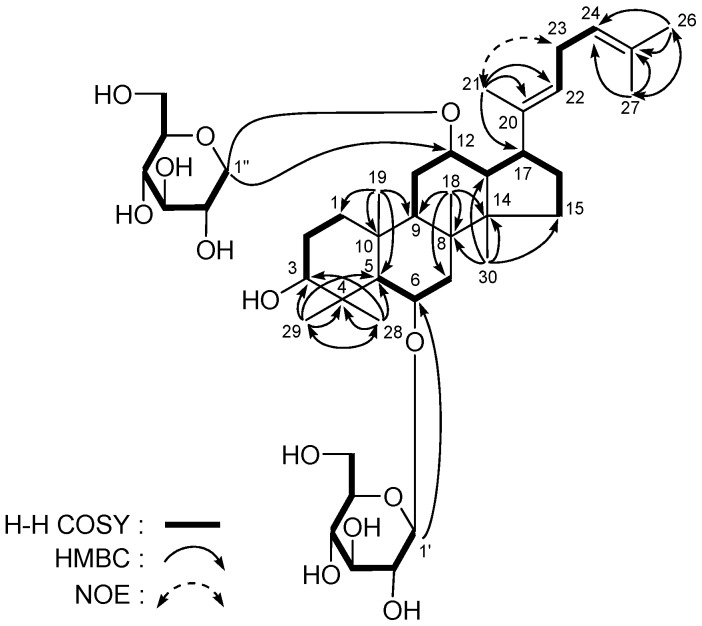
The main ^1^H ^1^H COSY, HMBC and NOE correlations of **7**.

*Sanchirhinoside B* (**7**), [*α*]^25^_D_ + 14.7° (MeOH), was isolated as a white powder. The molecular formula, C_42_H_70_O_13_, of 7 was determined by positive-ion HRESI-TOF-MS (*m/z* 805.4700 [M + Na]^+^, calcd. for C_42_H_70_O_13_Na 805.4709). The ^1^H-, ^13^C-NMR (C_5_D_5_N, Table 8) and various 2D NMR experiments, including ^1^H ^1^H COSY, HSQC, and HMBC of 7 suggested the presence of eight methyls, two olefinic protons, three methines bearing oxygen functions, together with two anomeric proton signals, which indicated that 7 was a dammarane-type triterpene saponin derivative with two double bonds. Comparison of the ^1^H- and ^13^C-NMR spectra of 7 with those of ginsenoside Rh_4_ [10] indicated that the two compounds had the same C-17 side chain. The stereochemistry of the double bond at C-20(22) was determined by a NOESY experiment. In the NOESY spectrum for 7, the correlation signal between *δ*_H_ 1.77 (3H, s, H_3_-21) and *δ*_H_ 1.74, 2.81 (1H each, both m, H_2_-23) was observed (Figure 5). Consequently, the configuration of double bond at C-20(22) was supposed to be *E*. Furthermore, in HMBC experiment, long-range correlations were observed between *δ*_H_ 5.01 (H-1') and *δ*_C_ 80.0 (C-6); *δ*_H_ 4.98 (H-1'') and *δ*_C_ 77.1 (C-12). Finally, acid hydrolysis of 7 only liberated D-glucose [7,8]. Therefore, the structure of 7 was concluded to be sanchirhinoside B as shown in Figure 5.

**Table 8:** ^1^H- and ^13^C-NMR data for compound **7** in C_5_D_5_N (500 MHz for ^1^H and 125 MHz for ^13^C).

No.	*δ*_C_	*δ*_H_ (*J* in Hz)	No.	*δ*_C_	*δ*_H_ (*J* in Hz)
1	39.2	0.88 (m), 1.51 (m)	23	27.8	1.74 (m), 2.81 (m)
2	28.0	1.84 (m)	24	124.8	5.41 (t, 7.0)
3	78.6	3.52 (dd, 5.0, 11.0)	25	130.4	—
4	40.4	—	26	25.9	1.71 (s)
5	61.5	1.37 (d, 10.5)	27	17.9	1.62 (s)
6	80.0	4.38 (m)	28	31.8	2.06 (s)
7	45.1	1.88 (m), 2.47 (m)	29	16.3	1.59 (s)
8	41.2	—	30	16.9	0.73 (s)
9	50.4	1.43 (m)	1'	105.9	5.01 (d, 7.5)
10	39.8	—	2'	75.5	4.09 (dd, 7.5, 8.0)
11	28.1	1.22 (m), 2.15 (m)	3'	79.7	4.24 (dd, 8.0, 9.0)
12	77.1	4.12 (m)	4'	72.5	4.11 (dd, 8.0, 9.0)
13	48.9	1.98 (dd, 10.5, 10.5)	5'	77.9	3.98 (m)
14	51.1	—	6'	63.1	4.37 (dd, 5.0, 12.0)
15	32.7	1.10 (m), 1.66 (m)			4.51 (dd, 2.0, 12.0)
16	29.4	1.44 (m), 1.78 (m)	1''	101.2	4.98 (d, 7.5)
17	49.6	2.77 (m)	2''	75.3	3.90 (dd, 7.5, 8.0)
18	17.2	1.09 (s)	3''	78.6	4.27 (dd, 8.0, 9.0)
19	17.7	0.91 (s)	4''	71.8	4.22 (dd, 9.0, 9.0)
20	138.4	—	5''	78.2	3.94 (m)
21	13.6	1.77 (s) 3.63 (1H, m, overlapped)	6''	63.7	4.36 (dd, 5.0, 12.0)
22	123.4	5.55 (t, 7.0)			4.59 (dd, 2.0, 12.0)

Furthermore, the protective effects of *P. notoginseng* 70% EtOH extract and new compounds 1–7 against antimycin A-induced mitochondrial oxidative stress were determined. The 70% ethanolic extract and compounds 4, 6 and 7 showed significant protective effects against antimycin A-induced L6 cell injury (Table 9).

**Table 9:** Cell survival rate of *P. notoginseng* extract and compounds **1**–**7** on L6 cells treated with antimycin A.

Sample	Cell survival rate (%)
Normal	100.0 ± 0.0 **
Control	45.9 ± 0.1
Probucol	56.1 ± 1.1 **
*P. notoginseng* ext.	55.3 ± 1.2 *
**1**	50.8 ± 1.9
**2**	56.8 ± 2.5
**3**	54.2 ± 1.5
**4**	59.3 ± 2.1 *
**5**	57.2 ± 3.1
**6**	59.0 ± 2.1 *
**7**	57.4 ± 1.6 *

Values represent the mean ± SD of determinations (*n* = 8). * *p* < 0.05; ** *p* < 0.01 *vs*. control group. Administrated concentration of probucol and **1**–**7** were 10 μmol/L, *P. notoginseng* ext. was 10 μg/mL. N = 8.

## 3. Experimental

### 3.1. General

Optical rotations were measured on a Rudolph Autopol^®^ IV automatic polarimeter. IR spectra were recorded on a Varian 640-IR FT-IR spectrophotometer. UV spectra were obtained on a Varian Cary 50 UV-Vis spectrophotometer. NMR spectra were determined on a Bruker 500 MHz NMR spectrometer at 500 MHz for ^1^H- and 125 MHz for ^13^C-NMR, with TMS as an internal standard. Positive- and Negative-ion HRESI-TOF-MS were recorded on an Agilent Technologies 6520 Accurate-Mass Q-Tof LC/MS spectrometer. Column chromatographies were performed on macroporous resin D101 (Haiguang Chemical Co., Ltd., Tianjin, China), silica gel (48–75 μm, Qingdao Haiyang Chemical Co., Ltd., Qingdao, China), Sephadex LH-20 (Ge Healthcare Bio-Sciences, Uppsala, Sweden), and ODS (40–63 μm, YMC Co., Ltd., Tokyo, Japan). A Cosmosil 5C18-MS-II (20 mm i.d. × 250 mm, Nakalai Tesque, Inc., Tokyo, Japan) preparative HPLC (PHPLC) column was used to purify the constituents. TLC plates pre-coated with silica gel GF_254_ (Tianjin Silida Technology Co., Ltd., Tianjin, China) were used to detect the purity of isolates by spraying with 10% aqueous H_2_SO_4_-EtOH, followed by heating.

### 3.2. Plant Material

The dried roots of *P.* *notoginseng* (Burkill, F. H. Chen) were collected from Wenshan, Guangxi province, China and identified by Dr. Li Tianxiang. The voucher specimen was deposited at the Academy of Traditional Chinese Medicine of Tianjin University of TCM (No. 20120505).

### 3.3. Extraction and Isolation

The dried roots of *P. notoginseng* (5.0 kg) were refluxed twice with 70% ethanol-water (volume) for 2 times. Evaporation of the solvent under reduced pressure provided a 70% ethanol-water extract (480.2 g). The residue was dissolved in H_2_O, then subjected to D101 CC [EtOH-H_2_O (0:100 → 50:50 → 100:0, v/v) to afford three fractions (Fr. 1−3). Fraction 3 (120.0 g) was subjected to silica gel CC [CHCl_3_ → CHCl_3_-MeOH (100:3 → 100:7, v/v) → CHCl_3_-MeOH-H_2_O (10:3:1 → 7:3:1 → 6:4:1, v/v/v, lower layer)] to give 12 fractions (Fr. 1–12). Fraction 7 (8.0 g) was subjected to normal phase silica gel CC [CHCl_3_ → CHCl_3_-MeOH-H_2_O (40:3:1 → 30:3:1 → 20:3:1 → 10:3:1, v/v/v, lower layer) → MeOH] to yield fourteen fractions (Fr. 7-1-1–7-1-14). Fraction 7-6 (97.9 mg) was purified by prepared HPLC (PHPLC) [MeOH-H_2_O (70:30, v/v)], and sanchirhinoside A_1_ (**1**, 2.9 mg) was obtained. Fraction 8 (4.0 g) was isolated by ODS CC [MeOH-H_2_O (40:60 → 50:50 → 60:40 → 70:30 → 80:20 → 100:0, v/v] to give 11 fractions (Fr. 8-1–8-11). Fractions 8-5 (46.6 mg), 8-6 (80.5 mg), and 8-8 (40.2 mg) were purified by PHPLC [MeOH-H_2_O (60:40, v/v)] to yield sanchirhinosides A_4_ (**4**, 1.6 mg), B (**7**, 3.3 mg), and A_2_ (**2**, 7.7mg), respectively. Fraction 9 (16.0 g) was subjected to ODS CC [MeOH-H_2_O (30:70 → 40:60 → 50:50 → 60:40 → 70:30 → 100:0, v/v)] to afford nine fractions (Fr. 9-1–9-9). Fraction 9-7 (113.8 mg) was purified by PHPLC [MeOH-H_2_O (60:40, v/v)], and sanchirhinoside A_3_ (**3**, 7.6 mg) was obtained. Fraction 10 (3.6 g) was separated by ODS CC [MeOH-H_2_O (10:90 → 20:80 → 30:70 → 40:60 → 50:50 → 60:40 → 70:30 → 80:20 → 100:0, v/v)] to afford 15 fractions (Fr. 10-1–10-15). Fraction 10-7 (393.8 mg) was purified by PHPLC [MeOH-H_2_O (50:50, v/v)] to give sanchirhinoside A_5_ (**5**, 8.2 mg). Fraction 12 (10.0 g) was subjected to ODS CC [MeOH-H_2_O (10:90 → 20:80 → 30:70 → 40:60 → 50:50 → 60:40 → 100:0, v/v)] to give 13 fractions (Fr.12-1–12-13). Fraction 12-9 (107.8 mg) was further purified by silica gel CC [CHCl_3_-MeOH-H_2_O (7:3:1, v/v/v, lower layer) to yield sanchirhinoside A_6_ (**6**, 12.7 mg).

*20S-Sanchirhinoside A_1_* (**1**): White powder. [*α*]_D_^25^ + 12.6° (*c* = 0.12, MeOH); IR ν_max_ (KBr) cm^−1^: 3,365, 2,928, 2,872, 1,717, 1,654, 1,457, 1,375, 1,316, 1,188, 1,085, 1,045. ^1^H-NMR (500 MHz, C_5_D_5_N) and ^13^C-NMR (125 MHz, C_5_D_5_N) spectroscopic data, see Table 1; ^1^H-NMR (500 MHz, CD_3_OD) and ^13^C- NMR (125 MHz, CD_3_OD) spectroscopic data, see Table 2. HRESI-TOF-MS: Positive-ion mode *m/z* 729.4543 [M + Na]^+^ (calcd’ for C_40_H_66_O_10_Na 729.4548); Negative-ion mode *m/z* 741.4364 [M + Cl]^−^ (calcd for C_40_H_66_O_10_Cl 741.4350).

*20S-Sanchirhinoside A_2_* (**2**): White powder. [*α*]_D_^25^ +7.4° (*c* = 0.33, MeOH); IR ν_max_ (KBr) cm^−1^: 3,367, 2,931, 2,876, 1,733, 1,642, 1,456, 1,373, 1,242, 1,160, 1,075, 1,043. ^1^H NMR (500 MHz, C_5_D_5_N) and ^13^C-NMR (125 MHz, C_5_D_5_N) spectroscopic data, see Table 3. Positive-ion mode *m/z* 835.4832 [M + Na]^+^ (calcd. for C_43_H_72_O_14_Na 835.4814); Negative-ion mode *m/z* 847.4568 [M + Cl]^−^ (calcd. for C_43_H_72_O_14_Cl 847.4616).

*20S-Sanchirhinoside A_3_* (**3**): White powder. [*α*]_D_^25^ + 19.7° (*c* = 0.36, MeOH); IR ν_max_ (KBr) cm^−1^: 3,367, 2,927, 2,875, 1,647, 1,457, 1,386, 1,253, 1,074, 1,027. ^1^H-NMR (500 MHz, C_5_D_5_N) and ^13^C-NMR (125 MHz, C_5_D_5_N) spectroscopic data, see Table 4. Positive-ion mode *m/z* 793.4720 [M + Na]^+^ (calcd. for C_41_H_70_O_13_Na 793.4709); Negative-ion mode *m/z* 815.4779 [M + COOH]^−^ (calcd. for C_42_H_71_O_15_ 815.4798).

*20S-Sanchirhinoside A_4_* (**4**): White powder. [*α*]_D_^25^ + 23.2° (*c* = 0.08, MeOH); IR ν_max_ (KBr) cm^−1^: 3,366, 2,929, 2,872, 1,643, 1,457, 1,386, 1,255, 1,127, 1,073, 1,043. ^1^H-NMR (500 MHz, C_5_D_5_N) and ^13^C- NMR (125 MHz, C_5_D_5_N) spectroscopic data, see Table 5. Positive-ion mode *m/z* 793.4716 [M + Na]^+^ (calcd. for C_41_H_70_O_13_Na 793.4709); Negative-ion mode *m/z* 815.4468 [M + COOH]^−^ (calcd. for C_42_H_71_O_15_ 815.4798).

*20S-Sanchirhinoside A_5_* (**5**): White powder. [*α*]_D_^25^ + 105.3° (*c* = 0.41, MeOH); IR ν_max_ (KBr) cm^−1^: 3,367, 2,930, 2,875, 1,647, 1,457, 1,386, 1,310, 1,073, 1,042. ^1^H-NMR (500 MHz, C_5_D_5_N) and ^13^C- NMR (125 MHz, C_5_D_5_N) spectroscopic data, see Table 6. Positive-ion mode *m/z* 955.5248 [M + Na]^+^, calcd. for C_47_H_80_O_18_Na 955.5237); Negative-ion mode *m/z* 967.4950 [M + Cl]^−^ (calcd. for C_47_H_80_O_18_Cl 967.5039).

*20S-Sanchirhinoside A_6_* (**6**): White powder. [*α*]_D_^25^ + 3.1° (*c* = 0.55, MeOH); IR ν_max_ (KBr) cm^−1^: 3,367, 2,929, 2,878, 1,645, 1,456, 1,386, 1,307, 1,074, 1,043. ^1^H-NMR (500 MHz, C_5_D_5_N) and ^13^C-NMR (125 MHz, C_5_D_5_N) spectroscopic data, see Table 7. Positive-ion mode *m/z* 1117.5725 [M + Na]^+^, calcd. for C_53_H_90_O_23_Na 1117.5765); Negative-ion mode *m/z* 1093.5731 [M - H]^−^ (calcd. for C_53_H_89_O_23_ 1093.5800).

*Sanchirhinoside B* (**7**): White powder. [*α*]_D_^25^ + 14.7° (*c* = 0.12, MeOH); IR ν_max_ (KBr) cm^−1^: 3,367, 2,927, 2,874, 1,653, 1,457, 1,395, 1,151, 1,072, 1,024. ^1^H-NMR (500 MHz, C_5_D_5_N) and ^13^C-NMR (125 MHz, C_5_D_5_N) spectroscopic data, see Table 8. Positive-ion mode *m/z* 805.4700 [M + Na]^+^, calcd. for C_42_H_70_O_13_Na 805.4709); Negative-ion mode *m/z* 817.4518 [M + Cl]^−^ (calcd. for C_42_H_70_O_13_Cl 817.4510).

### 3.4. Acid Hydrolysis of **1**–**7**

A solution of new compounds **1**–**7** (each 1.5 mg) in 1 M HCl (1 mL) was heated under reflux for 3 h, respectively. The reaction mixture was neutralized with Amberlite IRA-400 (OH^−^ form) and removed by filtration. The aqueous layer was subjected to the HPLC analysis under the following condition, respectively: HPLC column, Kaseisorb LC NH_2_-60-5, 4.6 mm i.d. × 250 mm (Tokyo Kasei Co. Ltd., Tokyo, Japan); detection, optical rotation [Chiralyser (IBZ Messtechnik GMBH, Hannover, Germany)]; mobile phase, CH_3_CN-H_2_O (75:25, v/v); flow rate 1.0 mL/min. As results, D-xylose (from **2**, **6**), L-arabinose (from **3**–**5**), D-glucose (from **1**–**7**) and were confirmed by comparison of the retention times with the authentic samples [*t*_R_: 8.8 min (D-xylose), 10.2 min (L-arabinose), and 13.1 min (D-glucose), all of them showed positive optical rotations].

### 3.5. Mitochondrial Oxidative Stress Protect Effects Assay

Antimycin A was used to induce mitochondrial oxidative stress [11]. Briefly, L6 cells (Cell Resource Center, IBMS, CAMS/PUMC, Beijing, China) were plated at a density of 5 × 10^4^ cells/well in Dulbecco’s modified Eagle’s medium (DMEM, Thermo Scientific, UT, USA) supplemented with 10% calf serum (Thermo Scientific) in a 96-well plate and were incubated at 37 °C for 24 h. Cells were treated with or without 10 μmol/L sample DMSO solution (final DMSO concentration was 0.5%). One hour later, medium was removed and 100 μg/mL antimycin A (Sigma Co. Ltd, MO, USA) in 200 μL DMEM was added to each well, The MTT assay was performed 24 h later to detect the cell survival rate. Probucol was used as positive control. 

### 3.6. Statistical Analysis

Values are expressed as mean ± S.D. All the grouped data were statistically performed with SPSS 11.0. Significant differences between means were evaluated by one-way analysis of variance (ANOVA) and Tukey’s Studentized range test was used for post hoc evaluations. *p* < 0.05 was considered to indicate statistical significance.

## 4. Conclusions

Antimycin A is known to cause the leakage of superoxide radicals from cell mitochondria by inhibiting mitochondrial electron transport [12]. Compared with normal group, 100 µg/mL antimycin A induced significant L6 cell injury, while 10 µM probucol showed increased cell survival rate effects compared with the antimycin treated group. From the bioactive 70% EtOH extract of *P. notoginseng* roots, seven new protopanaxatriol type saponins, 20*S*-sanchirhinosides A_1_–A_6_ (**1**–**6**), and sanchirhinoside B (**7**) were obtained. Among the new compounds, **4**, **6** and **7** showed significant protective effects against antimycin A-induced L6 cell injury. This research will benefit investigation of trace bioactive chemical constituents of *P. notoginseng* root. On the basis of the activity screening results, further studies of the antioxidant mechanisms of compounds **1**–**7** are necessary. 
